# Assessing the Quantitative Performance of Atmospheric Solids Analysis Probe‐Mass Spectrometry

**DOI:** 10.1002/rcm.10112

**Published:** 2025-08-01

**Authors:** Alisha Henderson, Stephanie Rankin‐Turner, James C. Reynolds, Matthew A. Turner, Ashley Sage, David Douce, Mario Thevis, Liam M. Heaney

**Affiliations:** ^1^ School of Sport, Exercise and Health Sciences Loughborough University Loughborough UK; ^2^ Department of Chemistry University of Pittsburgh Pittsburgh Pennsylvania USA; ^3^ Department of Chemistry, School of Science Loughborough University Loughborough UK; ^4^ Waters Corporation Wilmslow UK; ^5^ Centre for Preventive Doping Research, Institute of Biochemistry German Sport University Cologne Cologne Germany

**Keywords:** accuracy, ambient ionisation mass spectrometry, ASAP‐MS, precision, quantitation

## Abstract

**Rationale:**

Atmospheric solids analysis probe‐mass spectrometry (ASAP‐MS) is an established ambient ionisation technique that allows for the direct and rapid analysis of samples without chromatographic separation. Consequently, applications that typically benefit from ambient ionisation approaches can achieve improved sample throughput and thus improved potential for in situ testing. Previous reports have contrasted in viewpoints on the ability for ASAP‐MS to provide reliable quantitative data. Critically, in‐depth data exploring the quantitative capabilities of ASAP‐MS are currently lacking.

**Methods:**

Here, a series of experiments were performed to assess the quantitative performance of ASAP‐MS using a proof‐of‐concept single analyte (caffeine) approach. Analytical precision, accuracy, linearity and sensitivity were investigated using numerous variables, including sample deposition method (i.e., directly placing the probe into the sample vs. pipetting the sample onto the probe) and deposition volume, as well as the presence of a series of different internal standard approaches.

**Results:**

The data acquired demonstrated that the use of a positive displacement pipette and an isotopically labelled (structure‐matched) internal standard provided an optimal approach for quantitative reliability, albeit at levels often below the standards set for traditional chromatography‐based quantitative assays. The investigations were performed across a concentration range of 50–5000 ng/mL. Whereas measurable responses were seen across the full range in most approaches, limitations in sensitivity were identified and reduced quantitative performance statistics were noted at concentrations below 1000 ng/mL.

**Conclusions:**

These experiments demonstrate, for the first time, a comprehensive investigation into the quantitative performance of ASAP‐MS using caffeine as the example analyte. These data offer insight into the current strengths and limitations of quantitative analyses using ASAP‐MS and aim to provide practical recommendations to optimise quantitative approaches using this technique.

## 1 Introduction

Atmospheric solids analysis probe‐mass spectrometry (ASAP‐MS) is a form of ambient ionisation mass spectrometry (MS) that offers the capability to introduce samples into the ion source, in solid or liquid form, with little or no pre‐preparation. More specifically, ASAP‐MS is performed through the application of a sample onto a glass capillary tube (via dipping or deposition by a pipette), which is then directly inserted into the ion source housing of the mass spectrometer. A stream of heated gas causes rapid thermal desorption of the analytes from the probe, followed by ionisation by corona discharge atmospheric pressure chemical ionisation (APCI). The ionised analytes are then directed into the MS inlet for analysis [1, 2, 3, 4]. This technique offers the opportunity to develop and apply analytical approaches that provide rapid spectral output. In addition, the absence of solvents, reduced vacuum and power requirements and the reduced size of ambient ion sources create the potential for transportable instrumentation to reduce the time, complexity and overall cost of chemical analyses. Consequently, fields in which high sample throughput is crucial could benefit from the application of ASAP‐MS. For example, clinical assays capable of analysing and diagnosing a patient in a short time scale would greatly benefit from the timely determination of patient conditions, as well as help to improve the overall patient flow in hospitals and healthcare settings, transforming the current capabilities for point‐of‐care testing [5]. Additionally, the direct and rapid nature of ASAP‐MS opens opportunities for deployable MS systems for the field analysis of samples, providing an additional analytical offering to complement large laboratory‐based instruments [4]. A pertinent example of this potential falls within forensic assessments. On‐site analyses for identification of seized materials could provide fundamental information to assist in directing a case towards certain lines of enquiry, subsequently leading to improved forensic information and potential evidence for court proceedings.

The recent milestone marking 20 years of ambient ionisation MS has seen quantitation discussed as a technical challenge across multiple ionisation approaches [4, 6]. To date, analyses using ASAP‐MS have predominantly been applied in a qualitative manner, whereby predetermined ions (and potentially in‐source derived ion fragments) have been assessed to understand the presence/absence of an analyte [7, 8, 9, 10, 11]. However, the capacity to rapidly analyse samples to provide reliable quantitative data would offer this technology as a powerful addition to the toolbox of the applied analytical scientist, particularly when coupled with the opportunities for transportable, on‐site assessments. Despite the obvious advantages of these rapid, quantitative approaches to various fields of analytical science, limitations with quantitation are regularly reported across ambient ionisation‐based techniques [12], with limitations typically considered to be due to the lack of analyte separation afforded by chromatographic techniques. These challenges have been investigated across a range of ambient ionisation techniques through the application of sample pretreatment methods such as microextraction [13, 14] and derivatisation [15], albeit these approaches naturally lead to a decrease in throughput. Although not yet widely adopted, the use of an internal standard (IS) for ambient ionisation techniques is being more frequently adopted to improve quantitative performance whilst aiming to maintain a high level of throughput. This has been achieved through the integration of an IS within the sample loading source, such as through a coated capillary using a designated IS standard introduction system [16, 17] or by directly adding the IS to the sample, which is to be analysed [18, 19].

Although quantitative analyses have been performed using ASAP‐MS and reported within the literature [20, 21], these works lack a comprehensive understanding and demonstration of the reliability and repeatability of quantitation‐based approaches for ASAP‐MS [2, 22]. Consequently, the extent to which ASAP‐MS can be exploited for quantitative analysis requires further assessment [3, 5, 12].

This work applied experiments aimed to assess the quantitative performance of ASAP‐MS in terms of sensitivity, accuracy and repeatability. This was done through the investigation applying a single analyte proof‐of‐concept approach. Aspects of quantitative performance were assessed across a range of experiments including differing formats of sample deposition methods, assessments across a range of concentrations and sample deposition volumes and the influence of the use of both a surrogate and matched IS comparators.

## 2 Experimental

### 2.1 Materials

Methanol (MeOH), acetonitrile (MeCN) and water (all LC‐MS grade) were purchased from Fisher Scientific (Loughborough, UK). Caffeine (1 mg/mL ± 0.05% in MeCN) was obtained from Waters Corporation (Milford, MA, USA). Caffeine‐(trimethyl‐^13^C_3_) (^13^C_Caff_; 1 mg/mL in MeOH, 99 atom%^13^C, 99%, certified reference material, Cerilliant) was purchased from Merck (Gillingham, UK), with melatonin (labelled as melatonine, 99%) and theobromine (99%) purchased from Fisher Scientific. Closed soda glass capillary tubes (Samco G119/32) were obtained from Fisher Scientific (100 mm length, 1.8 mm outer diameter).

### 2.2 ASAP‐MS Parameters and Sample Preparation Details

The analyses were performed using a RADIAN ASAP Direct Mass Detector (Waters Corporation). The system consisted of an ASAP ion source coupled with a single quadrupole mass detector. Analyte ionisation was performed by APCI in positive ion mode, with the system operating in performance setup (i.e., with a floor standing rotary pump). The source temperature was set to 150°C, the corona current set at 3 μA, and the N_2_ gas heater temperature set at 600°C. Data were analysed across four scan modes with increasing cone voltages of 15, 25, 35 and 50 V. This approach was used to mimic the standard approach performed on this instrumentation to allow further investigation of in‐source fragmentation patterns; however, only the 15 V cone voltage data have been assessed for these experiments. Each scan mode was carried out with a mass range of *m/z* 50–600 in continuum mode, with a sampling frequency of 5 Hz. Additional data were collected on caffeine and ^13^C_Caff_ only using selected ion monitoring (SIM) mode for *m/z* 195.1 and 198.1, respectively. Samples were introduced into the RADIAN ASAP ion source using the dedicated RADIAN sample loading rig following deposit of the sample solution onto a closed soda glass capillary tube. Prior to use, the capillary tubes were preconditioned to remove any potential contamination using the default instrument protocol where they were placed into the N_2_ gas flow set to 600°C. Analyses of the data were performed using MassLynx 4.2 software (Waters Corporation) by extracting each ion of interest (see Table 1 for details) and integrating the smoothed extracted ion chromatogram peak area. For experiments utilising an IS approach, the extracted ion peak area of caffeine was normalised to the extracted ion peak area of each respective IS and the recalculated ratio data used for further assessment.

**TABLE 1:** Chemical properties of the analytes included within the experiments, detailing their chemical structure, monoisotopic mass and ion extraction criteria.

Analyte	Structure	Monoisotopic mass	Extracted ion (*m/z*)	Isolation window
Caffeine	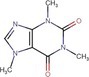	194.08038	195.1	0.4 Da
Caffeine‐(trimethyl‐^13^C_3)_	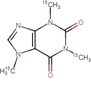	197.09044	198.1	0.4 Da
Melatonin	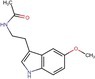	232.12118	233.1	0.4 Da
Theobromine	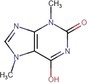	180.06473	181.1	0.4 Da

Caffeine was selected as the analyte to investigate the quantitative performance of ASAP‐MS due to its high ionisation affinity and known compatibility with APCI. This approach aimed to optimise peak extraction and repeatability to provide relevant indications of assay precision, linearity, accuracy and sensitivity. Caffeine calibration sets were prepared in water (herein referred to as aqueous), 50:50 MeOH:MeCN (herein referred to as organic) and 50:50 MeOH:MeCN containing ^13^C_3_‐caffeine, melatonin and theobromine each at 1000 ng/mL (herein referred to as the IS solution). The caffeine was diluted from a stock solution of 1 mg/mL to concentrations of 50, 200, 500, 1000, 2500 and 5000 ng/mL in each of the mentioned calibration solutions. Quantitative characteristics of the assays were assessed across a series of protocol variables including sample deposition method, sample deposition volume and MS scan parameters (for the isotopically labelled IS solution only). Precision data were determined by analysing each sample 10 times and comparing the repeatability across the analysis by calculating the relative standard deviation (%RSD) (i.e., level of imprecision) of the resultant extracted ion peak areas. Linearity assessments were performed by constructing calibration curves generated across the sample concentration range and analysing the data using linear regression. Accuracy values were assessed via a back‐calculation of each calibration point using the linear regression equation produced from the calibration curves. Finally, sensitivity was assessed by repeated 1:1 dilution of the lowest identified standard until a peak of the extracted ion with a signal‐to‐noise ratio (S:N) ≥ 10 could no longer be detected.

### 2.3 Investigating the Influence of Sample Deposition Method

Three different methods of depositing the sample onto the glass capillary tube were investigated in order to understand the influence these have on the quantitative performance of the ASAP‐MS system. These techniques included sample dipping, alongside the use of an air displacement pipette (ADP, Pipetman P10, 1–10 μL, Gilson, Middleton, MI, USA) and a positive displacement pipette (PDP, Microman E M10E, 1–10 μL, Gilson). Sample dipping was performed by submerging the glass capillary into the sample solution followed by loading into the ASAP source. In order to minimise sampling bias across analyses, the capillary was dipped into the sample at a consistent depth of 25 mm from the upper edge of a 1.5 mL autosampler vial containing a fixed volume (1 mL) of solution and held in situ for 20 s. A ruler was used to enable the same dipping depth of the capillary on each occasion. For pipetting‐based approaches, the glass capillary was placed onto the RADIAN loading rig, and the sample was deposited as a droplet onto the end of the glass before being immediately inserted into the ASAP source. For all sample analyses, the glass capillary was allowed to cool outside of the ion source for at least 20 s prior to sample deposition.

### 2.4 Assessing the Impact of Sample Deposition Volume

For analyses performed using a pipette‐based sample deposition, further investigations were completed to assess the impact of deposition volume on quantitative performance characteristics. For both ADP and PDP approaches, deposition volumes of 2, 4, 6, 8 and 10 μL were investigated.

### 2.5 Evaluating the Use of an IS

To understand how the inclusion of an IS affects quantitative performance, both non‐matched (i.e., surrogate compound) and matched (i.e., isotopically labelled) IS were analysed in parallel to the target caffeine analyte. For this investigation, the caffeine sample solutions were prepared as described previously. The assessment of both matched and non‐matched IS was performed to allow the comparison of quantitative performance using the gold‐standard method of including an isotopically labelled standard against a more cost‐effective approach of non‐matched, less costly chemicals. Melatonin was chosen as a non‐matched IS, as previous work has been performed using caffeine as the IS for melatonin quantitation [20]. In addition, theobromine was included due to its structural similarity as a methylated metabolite of caffeine, with the presence of a single additional methyl group at position 1 of the xanthine ring. The solutions were analysed using the three different sample deposition methods and across all deposition volumes for pipette‐based analyses.

## 3 Results and Discussion

### 3.1 Sample Deposition Method

**FIGURE 1:**
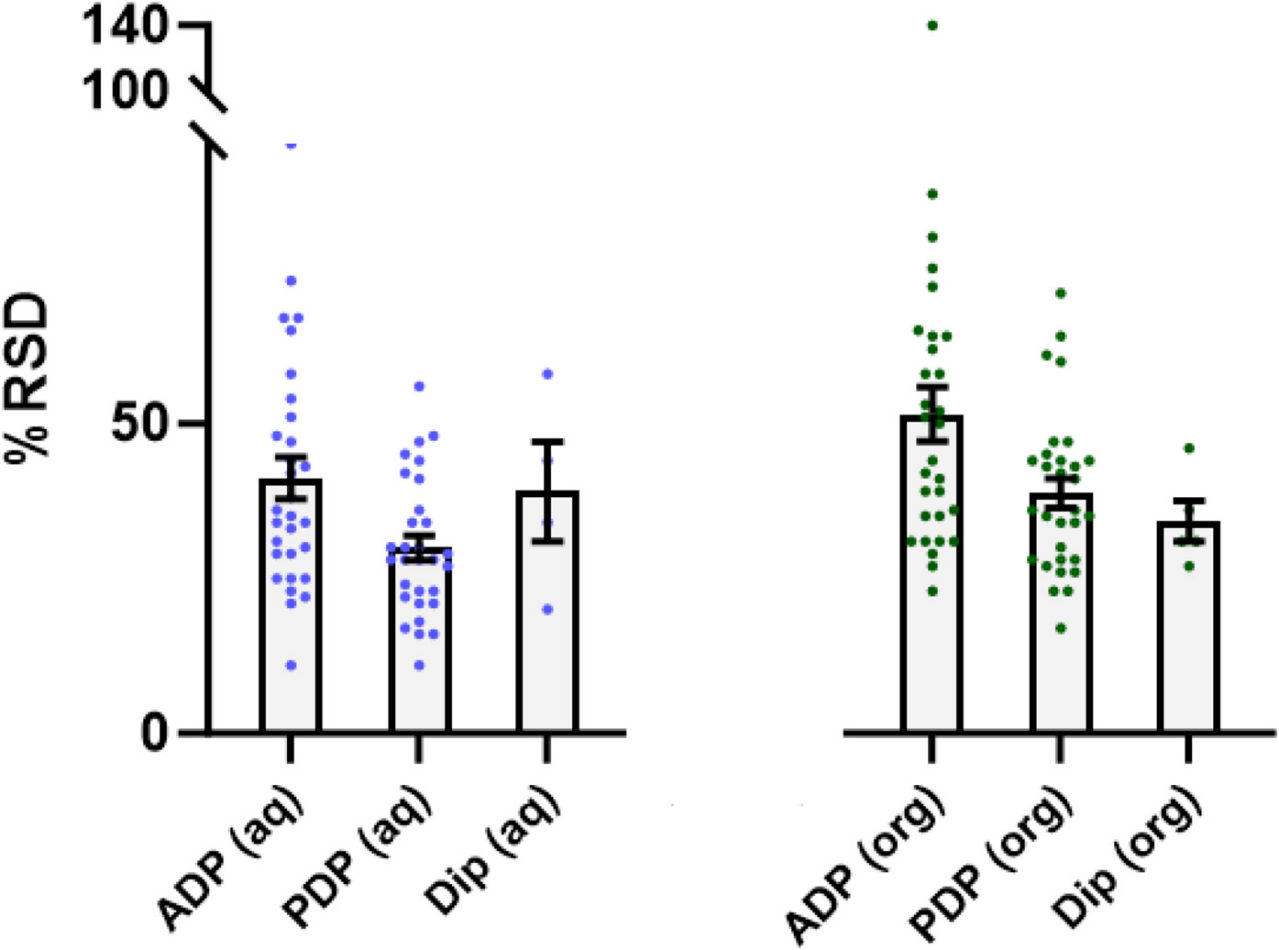
A column chart to show relative standard deviation (%RSD) values for repeated atmospheric solids analysis probe‐mass spectrometry analysis of caffeine in aqueous (aq, H_2_O) and organic (org, 50:50 MeOH:MeCN) solvent matrices. The %RSD was calculated for three different sample deposition methods including an air displacement pipette (ADP), a positive displacement pipette (PDP) and dipping the glass capillary tube into the sample (Dip). Data are taken across a concentration range of 5–5000 ng/mL, and the pipette‐based methods include data across all sample deposition volumes (2–10 μL). The columns indicate the average value, and error bars show the standard error of the mean.

In order to better understand the influence of sample deposition method on analytical precision, the %RSD values calculated from 10 repeated analyses of the caffeine standards across a range of concentrations, solvent solutions and deposition methods were compared (Figure 1). Taking into account the data for all measurements across the full range of volumes and concentrations, there was a clear indication that the use of a PDP improved the repeatability of analyses over an ADP (mean %RSD 29% [aqueous] and 39% [organic] vs. 41% and 52%, respectively; see Tables S1–S5 for expanded data). These data provide useful information for the application of ASAP‐MS as the comparison between different formats of micropipette models has not previously been completed with respect to the assessment of precision. The use of a PDP likely affords a greater level of precision due to direct contact between the piston and the sample within the pipette tip, allowing greater reproducibility for both the uptake and deposition of liquid over the use of an air displacement approach. Therefore, it is suggested that a PDP approach is used for sample deposition onto the glass capillary wherever possible.

Interestingly, and perhaps unexpectedly, the mean %RSD values recorded in this experiment for the dipping approach did not deviate notably from those recorded using the PDP method (mean %RSD 39% and 34% for aqueous and organic solvent matrices, respectively; see Table S6). These data do not match those seen previously within the literature, with one study demonstrating that much improved precision could be attained when using a pipette deposition over a dipping method [11]. This was determined in their investigation by a %RSD of < 15% when pipetting using an ADP versus < 50% for dipping. The authors attributed this outcome to the less defined/controllable protocols used for sample dipping, causing a greater variation in signal when compared to pipetting a fixed volume onto the capillary. For the current data, the improvement seen by Arrizabalaga et al. [11] for ADP over dipping were not replicated, with a modest improvement seen in the dipped experiments when compared to the ADP data (mean %RSD 34% vs. 51%, respectively, from the organic solvent analyses). Importantly, this experiment employed a strict and defined protocol for dipping to minimise sampling bias and maintain sample loading characteristics as consistent as possible. This included strict definitions for the depth of the glass capillary tube when placed within the sample vial, as well as the time the glass capillary tube remained submerged. This more defined dipping approach may decrease the level of sample‐to‐sample deviation seen in previous reports. However, it must be noted that as the sample volume cannot be altered/set during dipping (i.e., it is not strictly controlled), fewer sample analyses were completed overall when compared to pipette‐based methods, meaning the extent of the variation seen from dipping may not have been fully identified, especially considering that lower concentration analyses were not always possible (i.e., no caffeine ion detected). Nonetheless, these data show the potential for comparatively reproducible analyses with a dipping approach compared to pipette deposition, providing a strict dipping protocol is in place. This could, in turn, hold practical advantages for potential on‐site analyses, where a dipping sample loading protocol provides a simple, rapid and non‐specialist approach.

In conclusion, these data demonstrate that the use of PDP pipette offers the greatest repeatability to ASAP‐MS analyses, with the potential to use a dipping method where it may be advantageous, providing a strict sampling protocol is followed.

### 3.2 Sample Deposition Volume

When considering the use of a pipette‐based deposition method, it is beneficial to understand the impact that deposition volume has on assay precision. To this note, the use of deposition volumes in the literature has not been consistent between investigations where pipette‐based deposition of samples for ASAP‐MS analysis has been performed. For example, 2 μL depositions have been used on multiple occasions [11, 23], as well as the implementation of 5 or 7 μL as the deposited volume [11, 24]. These varied decisions on the level of deposition demonstrate the applicability to assess volumes ranging from 2 to 10 μL in this study, with further information gained by comparing across micropipette systems (i.e., ADP vs. PDP).

**FIGURE 2:**
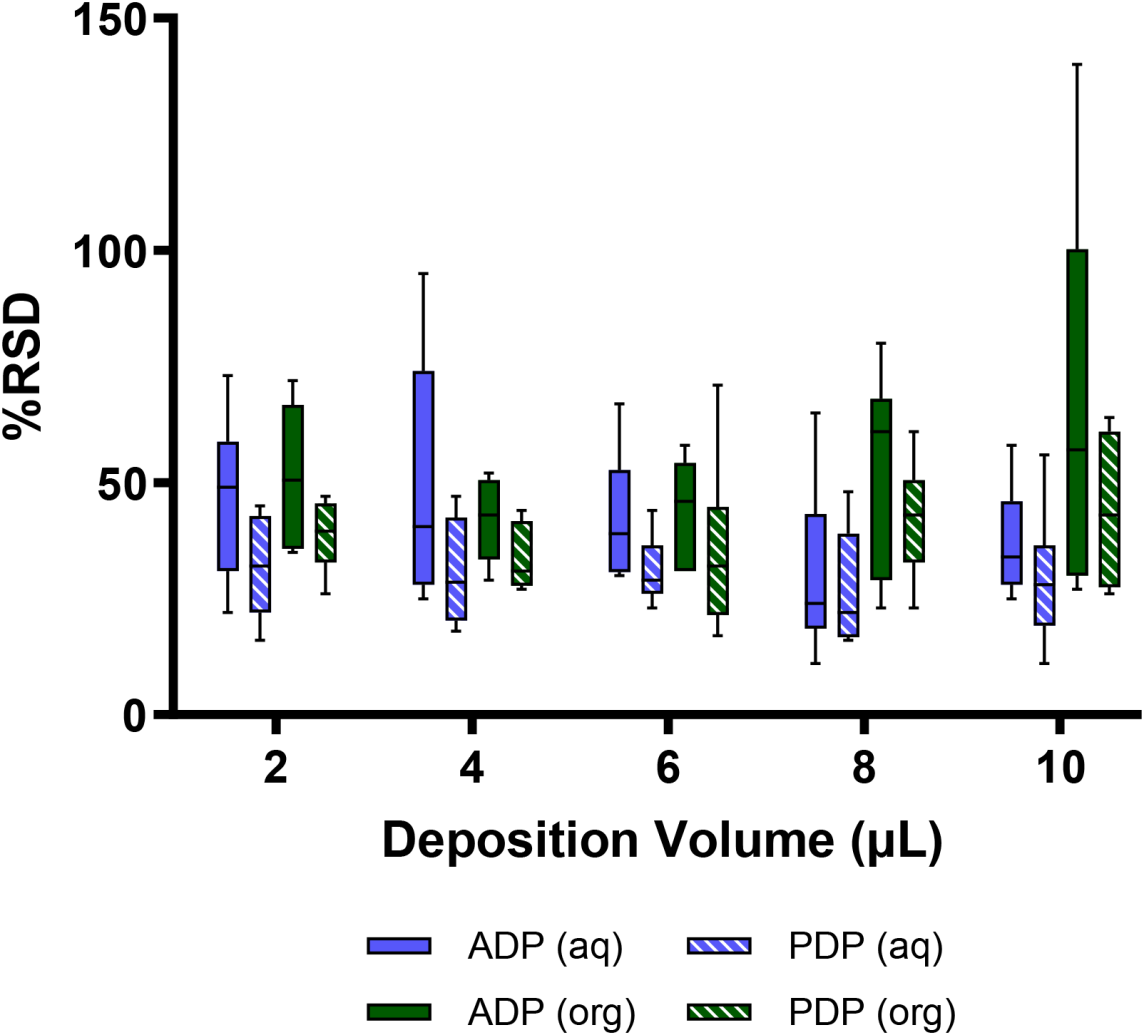
A box‐and‐whisker plot to show the impact of sample deposition volume on assay precision for atmospheric solids analysis probe‐mass spectrometry analyses of caffeine in aqueous (aq, H_2_O) and organic (org, 50:50 MeOH:MeCN) solvent solutions. Mean relative standard deviation (%RSD) values were calculated for differing sample deposition volumes and combined across a concentration range of 50–5000 ng/mL. Samples were analysed using an air displacement pipette (ADP) or a positive displacement pipette (PDP) for comparison. The boxes indicate the median and interquartile range, and whiskers show the range.

Although, in general, it could be considered that a larger volume droplet deposited onto the ASAP glass capillary tube would increase the ion response and thus improve precision, it must also be considered that the size of the droplet (i.e., larger volume gives a larger droplet) could impact the efficiency of sample delivery into the ion source and its subsequent desorption characteristics. Data from the pipette comparison experiment (Figure 1) showed that PDP, on average, improves precision over the use of ADP. When these data are split out by sample deposition volume, PDP use improves analytical repeatability when comparing the full calibration range across all sample deposition volumes (50–5000 ng/mL, 2–10 μL; Figure 2). The %RSD values for individual concentrations across the range can be found in Tables S1–S5. For the analyses performed using the aqueous solvent, precision was similar across deposition volumes for PDP (mean %RSD range for 2–10 μL; 27%–32%), whereas a small improvement in repeatability could be seen in higher volumes when using ADP (mean %RSD 2 μL vs. 10 μL; 47% vs. 37%). This suggests that precision remains relatively consistent irrespective of deposition volume when using a PDP approach. When comparing PDP to ADP, the biggest improvements in precision were identified at the lower volumes (mean %RSD PDP vs. ADP; 2 μL 32% vs. 47% and 4 μL 31% vs. 49%), with similar mean RSD values noted between pipette formats at the higher volumes (mean %RSD PDP vs. ADP; 8 μL 27% vs. 30% and 10 μL 29% vs. 37%). Overall, a similar profile was observed for the organic solvent analyses when comparing PDP to ADP. However, in the organic ADP analyses, it appeared that analytical precision worsened as deposition volume increased (mean %RSD 2 μL vs. 10 μL; PDP 39% vs. 44% and ADP 52% vs. 67%). It is not directly clear the reasoning for this observation, although it is possible that the lower viscosity of the organic solvent mixture is causing small losses of sample liquid from the glass capillary in the period between sample deposition and sample desorption (i.e., loss of sample during the insertion of the probe into the source). It is considered unlikely that sample evaporation is driving any variability. This is due to the consideration that total analyte load within the source is likely to be the same as caffeine would be retained in the remaining solvent/on the capillary.

From the data visualised in Figure 2, it is suggested that a sample deposition volume of 4 μL is used in conjunction with a PDP loading approach. Although the 4 μL experiments do not show the overall lowest median values for precision (e.g., the 8 μL PDP experiment has a median %RSD of 27%), the PDP data for both the aqueous and organic experiments demonstrate median precision values towards the lower end identified, as well as generally reduced overall ranges of precision compared to other recorded volumes. The investigation on the effect of sample volume deposition demonstrates applicability to support a range of different volume‐driven solvent extraction protocols to be used in the field. Although the presented data are related specifically to a single analyte in a particular concentration range, smaller deposition volumes could be advantageous to ASAP‐MS applications where solvent extraction may be required from limited sample content, for example, forensic investigations. Additionally, clinical applications of ambient MS that use a smaller sample volume may improve the ease of sampling and wider applicability of the method, for example, when using microsampling techniques for blood analyses [22]. Nonetheless, other analytical protocols may benefit from the use of larger sample deposition volumes. For example, applications where lower concentrations of analyte are present, such as pharmaceutical contamination or drug/toxin analyses, may require a larger sample deposition volume to increase the in‐source mass of the analyte to ultimately increase signal intensity and thus detection/quantitative performance. Therefore, this demonstrates the potential advantage for use of a range of sample deposition volumes to best understand the impact on quantitative performance of the assay. However, the ultimate range of sample deposition volumes possible with ASAP‐MS are limited to those that are feasible for retention on the glass capillary rod prior to insertion into the ion source. Future optimisation and validation of quantitative ASAP‐MS approaches should assess sample deposition volume to ensure the volume is suitable for the given application.

### 3.3 Inclusion of an IS

#### 3.3.1 Precision

**FIGURE 3:**
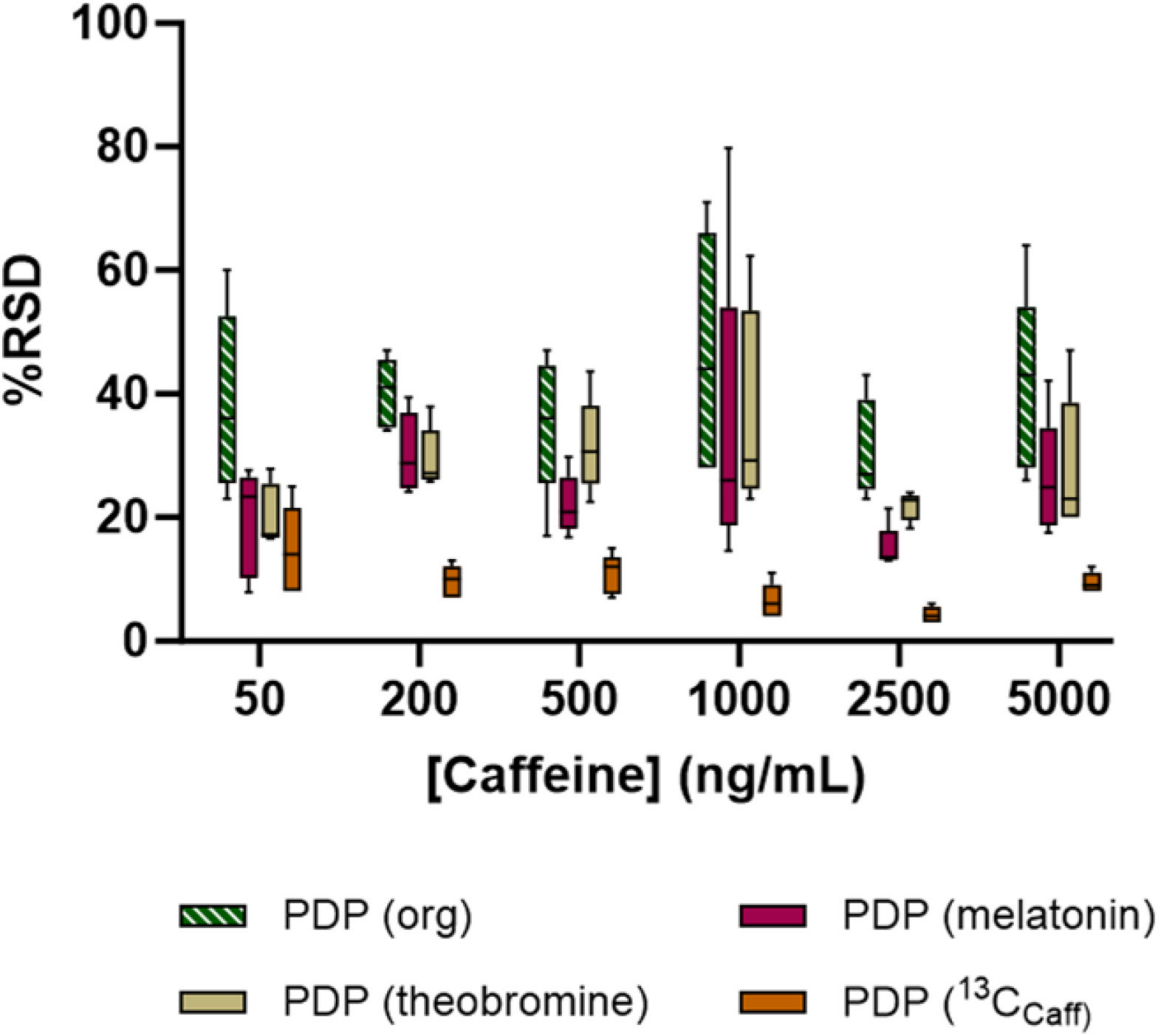
A box‐and‐whisker plot to show analytical precision when including a non‐matched surrogate (melatonin), a similar structured surrogate (theobromine) and a stable isotope label match [caffeine‐(trimethyl^13^C), ^13^C_Caff_] internal standard. Mean relative standard deviation (%RSD) was calculated and compared for caffeine responses normalised to the internal standard responses prepared in an organic (50:50 MeOH:MeCN) solvent solution and measured across the concentration range of 50–5000 ng/mL using deposition volumes of 2, 4, 6, 8 and 10 μL via a positive displacement pipette (PDP).

In order to assess the value of including an IS within the ASAP‐MS quantitative analyses, additional caffeine solutions were prepared as described previously, with the addition of three IS compounds at a concentration of 1000 ng/mL each in the organic solvent mixture. The IS included a non‐matched surrogate compound (melatonin), a similarly structured non‐matched surrogate (theobromine, a metabolite of caffeine) and a stable isotope labelled match (^13^C_Caff_). An overview of the %RSD values recorded when applying each of the different IS approaches using the PDP pipetting method can be seen in Figure 3. For melatonin, the %RSD was higher than ^13^C_Caff_ across all sample volumes and pipette methods (mean %RSD range melatonin vs. ^13^C_Caff_; ADP 19%–45% vs. 8%–27% and PDP 15%–34% vs. 8%–22%; see Tables S7–S16 for expanded data). Although this is expected, due to the higher structural similarity of a matched/labelled IS in comparison to a non‐matched surrogate, it was interesting to note that melatonin obtained similar %RSD values when compared to theobromine (mean %RSD range melatonin vs. theobromine; ADP 19%–45% vs. 24%–45% and PDP 15%–34% vs. 20%–32% across 50–5000 ng/mL). As theobromine is closely matched in structure to caffeine, it was expected that it could offer use as an IS that would provide improvements in assay precision closer to those seen with the labelled ^13^C_Caff_. However, the data show that no benefits for the use of theobromine were seen over the use of a non‐related surrogate compound (melatonin). Despite the inability for melatonin to offer a cost‐effective and simple IS approach, the data follow those identified in an interlaboratory study conducted by the British Mass Spectrometry Society (BMSS) [12]. In particular, the interlaboratory study demonstrated that even a non‐matched IS still provided an improved level of repeatability when compared to using the analyte peak response in isolation, with this characteristic repeated within the present experiments (mean %RSD range for melatonin IS vs. caffeine in organic solvent; ADP 19%–45% vs. 33%–69% and PDP 15%–34% vs. 31%–47% across 50–5000 ng/mL). Consequently, it is evident that a non‐matched IS can still improve quantitative assay precision, albeit at a modest level. This outcome of improved precision using either a labelled or non‐labelled IS has also been noted in AIMS imaging techniques. For example, experiments using nano‐DESI imaging noted that experiments using only an external standard generally underestimated sample concentrations, with similar levels of improved precision noted for the inclusion of any format of IS (non‐labelled %RSD 36%; labelled RSD 47%) [25]. The greatest benefits to assay precision from the current experiment were achieved through the addition of ^13^C_Caff_ as the IS. This was also previously reported via the BMSS interlaboratory study where a labelled IS provided an assay precision at 10%, a value similar to the highest levels of precision seen when using ^13^C_Caff_ in this experiment (%RSD of ~8%). Notably, the use of the labelled IS provided improvements in assay precision of around 20% over analyses performed with no IS comparator (mean %RSD range ^13^C_Caff_ vs. organic solvent; PDP 8%–22% vs. 31%–47%), with the values below 10% being noted at a concentration of 1000 ng/mL and above.

Of note, the use of the labelled IS was also able to appreciably improve assay precision when using the dipping approach (%RSD range when dipping with ^13^C_Caff_ vs. organic solvent across 500–5000 ng/mL, 17%–32% vs. 27%–36%; see Table S17 for individual %RSD values across the analysed ranges). These results demonstrated similar values to those that have previously been reported using ASAP‐MS for the analysis of drugs of abuse, with %RSD values of 13%–39% obtained when using a matched IS (%RSD in this experiment ranged from 16% to 32%) [19], as well as for the measurement of steroid esters (%RSD values < 20%) [26]. This shows agreement between multiple experiments that the repeatability of ASAP‐MS analyses can be maintained to a reasonable level when a controlled dipping approach is coupled with the use of a labelled IS. Previous experiments using alternative ambient ionisation MS approaches have also found benefit in the addition of a labelled IS compound. For example, in a study applying paper spray MS, the highest level of precision was observed at 5% [17], a similar value to that obtained in this experiment with 3% being observed for the 2500 ng/mL analysis using a PDP with a 4 μL deposition volume.

#### 3.3.2 Assay Linearity and Accuracy

In order to understand the capacity for the ASAP‐MS to produce linear regression equations suitable for quantitation, caffeine samples were analysed for linearity and back‐calculated accuracy across a range of 50–5000 ng/mL (200–5000 ng/mL for dipping) with and without the inclusion of the ^13^C_Caff_ IS. For the purpose of these experiments, an accurate result was determined as 100%.

Linearity of the calibration when using the dipping method was poor in the aqueous solution (*r* ^2^ = 0.6431), with a large improvement seen when using the organic solvent (*r* ^2^ = 0.9505; see Table S6). The reason for the poor performance using the aqueous solvent is not fully understood, but high values obtained at the 2500 ng/mL level (accuracy = 251%) coupled with low values reported at 500 and 5000 ng/mL (accuracy = 52% and 62%, respectively) drove this weak linear fit. This issue with linearity was negated to some extent using the organic solvent; however, accuracy values remained poor across the calibration range showing gross under‐reporting of concentrations (mean accuracy = 30%; range = 8%–45%). This demonstrated that the use of the dipping method in isolation was not able to provide reliable quantitative measurements. The inclusion of an IS when using the dipping approach varied in success. The addition of melatonin reduced the overall linearity of the experiment (*r* ^2^ = 0.8755) with poor accuracy (mean = 69%). Theobromine provided similar results to those without the inclusion of an IS (*r* ^2^ = 0.9684), again with poor accuracy (mean = 64%). However, the inclusion of ^13^C_Caff_ offered a high level of linearity (*r* ^2^ = 0.9969) and greatly improved the overall accuracy of the assay (mean = 101%; range = 86%–128%; see Table S17). These observations offer the potential for quantitative data to be obtained from dipping a sample into a liquid using a labelled and matched IS, albeit caveated with varying levels of analytical precision.

Patterns observed for pipetting experiments were variable, with deviations in levels in linearity seen across solvent matrices and pipette equipment (see Tables S1–S5); this is likely an artefact of inadequate analytical precision causing individual experiments to have increased variability. In general, a PDP approach (*r* ^2^ range = 0.9188–0.9885) provided more reliable data over the use of an ADP (*r* ^2^ range = 0.8332–0.9939); however, accuracy remained highly variable across all datasets including pipette equipment and deposition volume, with few calibration levels achieving an accuracy value of ±15% from the expected value (only 32% of all calibration levels investigated). This indicated a lack of reliability for quantitative data to be obtained via deposition using a pipette without the presence of an IS. Similarly to the dipping approach, the addition of melatonin as the IS did not improve the linearity of the calibration analyses (*r* ^2^ range = 0.8821–0.9271), with theobromine (*r* ^2^ range = 0.9596–0.9969) and ^13^C_Caff_ (*r* ^2^ range = 0.9956–0.9989) consistently outperforming melatonin with respect to assay linearity (see Tables S7–S16). Importantly, the inclusion of the isotopically labelled IS allows a consistent regression coefficient of > 0.99 to be obtained. When considering the accuracy of the back‐calculated data obtained from the calibration experiments, levels were considered generally acceptable (±20%) for all analyses of 1000 ng/mL and above for data obtained alongside ^13^C_Caff_ (range = 89%–118%), with values at 500 ng/mL often close to the ±20% target. This observation is in line with previous ASAP‐MS data where accuracy values were improved with inclusion of an IS (92.2% vs. 126.3% for inclusion and exclusion of an IS, respectively) [19]. This pattern was not replicated in the melatonin and theobromine experiments, which showed irregular variability across the calibration points. An example of a calibration curve from each of the deposition methods (applying the ^13^C_Caff_ IS) can be seen in Figure S1.

Overall, these experiments demonstrated that acceptable levels of precision and accuracy for ASAP‐MS analyses of caffeine could be obtained from around 1000 ng/mL upwards when using a pipetting deposition method including an isotopically labelled IS for signal normalisation. Although these parameters do not strictly match the validation parameters provided in bioanalytical guideline documents (e.g., precision and accuracy ±15%) across all levels [27, 28], there is clear potential for the further development of these characteristics for specific targeting of analytes of interest. It must be noted that the data presented in this work were generated using relatively generic instrument settings for a broad set of ASAP‐MS applications, meaning that there remains scope to further improve the quantitative performance through additional assay development. That said, the determination for the applicability/suitability of a reliable quantitative range should be considered on a case‐by‐case basis dependent on both the analyte of interest and the likely concentration of the sample being investigated. For example, an application for caffeine measurements could relate to the quality control/safety measurement of caffeinated energy drinks. Most energy drinks contain a reported caffeine level of around 320 μg/mL [29]; therefore, the knowledge of a suitable calibration range for ASAP‐MS would allow appropriate dilution to ensure quantitative performance with reduced risk of detector saturation and instrument contamination. On the contrary, if ASAP‐MS users are assessing target analytes that are known to be below the acceptable quantitative levels for the assay, information on relevant quantitative ranges can inform users on the requirement for aspects such as sample deposition volume and the potential requirement for pre‐concentration techniques to be employed, albeit at the cost of reduced throughput. Although these analyses show promise for quantitation using ASAP‐MS, it must be considered that these values are measured in a clean matrix and therefore may not translate to more complex matrices, such as biofluid samples, as well as achieving more stringent validation parameters outlined in bioanalytical guidance documents [27, 28].

### 3.4 The Influence of SIM Mode

**FIGURE 4:**
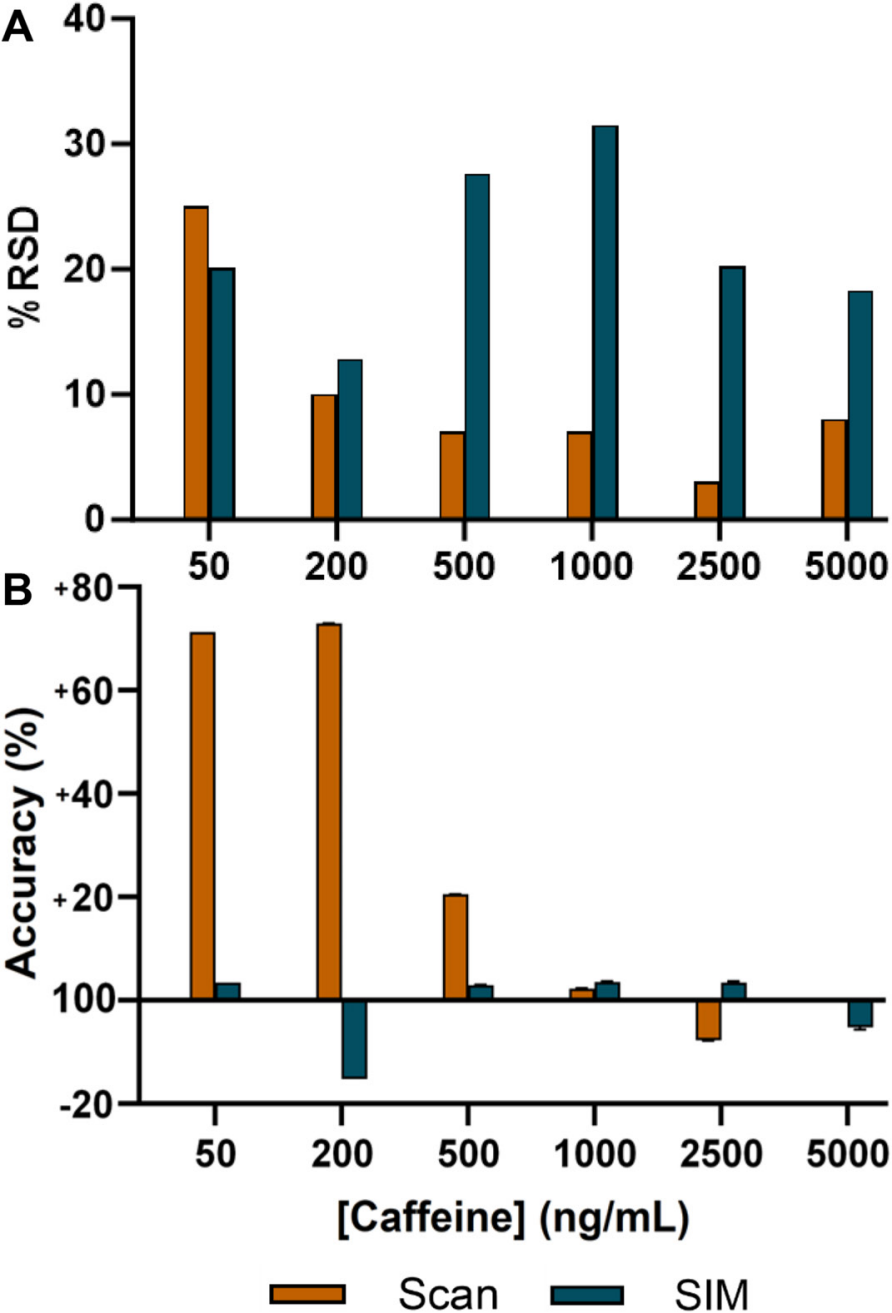
Comparison of (A) analytical precision and (B) accuracy for scan and selected ion monitoring (SIM) acquisition modes on the RADIAN ASAP‐MS system for the analysis of caffeine collected across a 50–5000 ng/mL calibration range. The analyses were performed with a 4 μL deposition using a positive displacement pipette to quantitate caffeine with ^13^C_Caff_ (1000 ng/mL) as an internal standard.

SIM mode utilises the detection of specific pre‐selected *m/z* values as opposed to completing a scanning acquisition across the full mass range. This, in turn, can improve the quality for both qualitative and quantitative data investigations. Critically, the reduced level of analysed *m/z* values allows for an increased dwell time of each of the pre‐selected ions, with ions outside of the pre‐selected *m/z* values being ejected from the mass analyser and thus not transferred to the detector. This mode of acquisition consequently allows the signal of the pre‐selected ions to be enhanced, alongside the reduction of the transmission for ions not relevant to the analyses, thereby offering tangible improvements in S:N for the ions/analytes of interest [30]. However, it must be noted that this selective ion approach occurs within the mass analyser, and, therefore, it cannot overcome issues with competitive ionisation occurring within the ion source itself. Importantly, SIM mode has shown to be a useful approach to help address issues with sensitivity that are inherent in ASAP‐MS and across a range of other ambient ionisation techniques. For example, in nano‐DESI imaging, SIM mode was shown to enhance the signal for low abundance species of interest and consequently improve the overall quality of the acquired imaging data [31, 32]. For ASAP‐MS, one investigation utilised the different modes of the mass analyser by using scan mode for the analysis of more readily abundant 30–150‐μm‐sized microplastics, with SIM mode applied to improve the limits of detection (thus increasing method sensitivity) to analyse less abundant 10‐μm‐sized microplastics [33]. For portable ambient ionisation techniques, the improved S:N can facilitate both qualitative and quantitative on‐site monitoring of targeted analytes in applications such as food or environmental testing [34, 35]. To further understand the impact of using a SIM mode approach on the quantitative performance with ASAP‐MS, one experiment was repeated using a two‐channel SIM method which targeted ions for caffeine (*m/z* 195.15) and ^13^C_Caff_ (*m/z* 198.17). The method applied a 4 μL deposition using a PDP with inclusion of the isotopically labelled IS. This was chosen due to the comparably acceptable levels of precision, accuracy and linearity observed across the calibration range of 500–5000 ng/mL. Interestingly, the analyses performed in SIM mode demonstrated an unexpected decrease in analytical precision when compared to scan mode comparators (Figure 4A). Whereas the mean %RSD was decreased in the SIM mode data at the lowest calibration point (50 ng/mL; 20% vs. 25% for SIM and scan, respectively), all other calibration levels showed a small (200 ng/mL; 13% vs. 10%) or marked (500–5000 ng/mL; 13%–32% vs. 3%–8%) decrease in analytical precision when SIM mode was used. A previous study that applied SIM mode using a larger footprint orbitrap‐MS setup suggested that increased presence of ions with similar *m/z* values afforded by SIM mode could adversely affect the quantitative performance of the instrument [36]. Therefore, the increased presence of similar ions (i.e., caffeine and ^13^C_Caff_) could have limited the detection characteristics and contributed to reduced analytical precision for the ASAP‐MS at higher concentrations.

Contrary to the performance recorded for analytical precision, experiments using SIM mode demonstrated meaningful increases in measurement accuracy. Whereas scan mode data demonstrated acceptable levels of accuracy in the 500–5000 ng/mL range (92%–121%), data collected via SIM mode reported excellent accuracy (±15%) across the full analytical range (50–5000 ng/mL; 85%–104%). This was greatly improved in a like‐for‐like comparison against the accuracy recorded in the initial full scan experiments (Figure 4B). This improvement in accuracy is likely an artefact of the reduced noise afforded by the SIM mode parameters, with only the ions of interest being measured. Although these data show mixed improvements in using SIM to quantitate an analyte of interest, it must be remembered that in a simple, clean matrix, there is little chance of interference and thus the exclusion of wider scan data when using a more complex matrix may not allow confidence that only the analyte of interest is contributing to the measured ions.

To further understand the impact of SIM on analyte detection, serial twofold dilutions of the caffeine standard were performed and analysed against a full scan comparator. In addition to the improvements in accuracy, SIM mode also provided increased analytical sensitivity, with a detectable caffeine peak (S:N > 10) at 6.25 ng/mL, with scan mode reporting the lower limit of detection at 25 ng/mL. This is as expected due to the decreased noise and increased dwell time given to the ions of interest when operating in SIM mode, improving the detection limit of the targeted analyte.

## 4 Conclusions

The present study evaluated quantitative performance using a single quadrupole‐based ASAP‐MS system. Variables including sample deposition approach and volume, the use of an internal standard and ion acquisition method were investigated to assess the subsequent influence on analytical precision, accuracy, linearity and sensitivity. This systematic approach has provided a more in‐depth understanding of the capabilities of the instrumentation for quantitative ASAP‐MS analysis. Overall, the use of a positive displacement pipette for sample deposition offered the greatest levels of analytical precision. Although precision and accuracy varied nominally when assessed across a range of sample deposition volumes, a loading volume of 4 μL, with the addition of an isotopically labelled (and compound matched) internal standard, offered the most repeatable and accurate results. Whereas the use of the isotopically labelled internal standard consistently improved values of precision, limitations in accuracy at the lower concentrations were still noted. Finally, applying a SIM acquisition mode provided improved sensitivity and accuracy when compared to analyses using a full scan approach, albeit with a trade‐off for decreased levels of measurement repeatability. Whereas this set of experiments offers valuable data and understanding to the field of quantitative ASAP‐MS, the demonstration of these data using a single analyte in clean matrices means that translation to other analyses should ensure the inclusion of initial investigations to understand the impact of the required concentration range and potential matrix confounders.

In conclusion, this manuscript provides a timely and thorough assessment of the quantitative capacity of a small form factor ASAP‐MS instrument. The data demonstrate the initial potential for ASAP‐MS to be used as a quantitative method in future investigations but highlight the variable nature of the analyses that have been generally overlooked in previous reports of quantitation using this approach. The data suggest generalised recommendations for the use of a positive displacement pipette at a mid‐level volume with the inclusion of a structure‐matched, isotopically labelled internal standard to maximise quantitative performance.

## Author Contributions

**Alisha Henderson:** conceptualization, methodology, investigation, writing – original draft, validation, visualization, formal analysis, data curation. **Stephanie Rankin‐Turner:** writing – original draft, writing – review and editing, supervision. **James C. Reynolds:** writing – review and editing, supervision. **Matthew A. Turner:** writing – review and editing, supervision. **Ashley Sage:** methodology, writing – review and editing, resources. **David Douce:** methodology, writing – review and editing, resources. **Mario Thevis:** supervision, writing – review and editing. **Liam M. Heaney:** conceptualization, writing – original draft, methodology, writing – review and editing, project administration, supervision, resources.

## Conflicts of Interest

The authors declare no conflicts of interest.

## Supporting information


**Table S1:** Precision, accuracy and linearity statistics at each concentration for caffeine analysis using atmospheric solids analysis probe‐mass spectrometry in aqueous and organic solvent matrices applying a 2 μL deposition with an air displacement (ADP) and positive displacement (PDP) pipette.
**Table S2:** Precision, accuracy and linearity statistics at each concentration for caffeine analysis using atmospheric solids analysis probe‐mass spectrometry in aqueous and organic solvent matrices applying a 4 μL deposition with an air displacement (ADP) and positive displacement (PDP) pipette.
**Table S3:** Precision, accuracy and linearity statistics at each concentration for caffeine analysis using atmospheric solids analysis probe‐mass spectrometry, in aqueous and organic solvent matrices applying a 6 μL deposition with an air displacement (ADP) and positive displacement (PDP) pipette.
**Table S6:** Precision, accuracy and linearity statistics at each calibration level using the dipping approach for caffeine analysis by atmospheric solids analysis probe‐mass spectrometry. Shaded boxes note concentrations at which no signal was detected.
**Table S7:** Precision, accuracy and linearity statistics at each concentration for caffeine analysis in organic solvent (50:50 MeOH:MeCN) with no inclusion of an internal standard (IS) and with melatonin, theobromine and caffeine‐(trimethyl‐13C3) (13CCaff) as an IS. A 2 μL deposition was made using an air displacement pipette (ADP).
**Table S8:** Precision, accuracy and linearity statistics at each concentration for caffeine analysis in organic solvent (50:50 MeOH:MeCN) with no inclusion of an internal standard (IS) and with melatonin, theobromine and caffeine‐(trimethyl‐^13^C3) (^13^CCaff) as an IS. A 2 μL deposition was made using a positive displacement pipette (PDP).
**Table S9:** Precision, accuracy and linearity statistics at each concentration for caffeine analysis in organic solvent (50:50 MeOH:MeCN) with no inclusion of an internal standard (IS) and with melatonin, theobromine and caffeine‐(trimethyl‐^13^C3) (^13^CCaff) as an IS. A 4 μL deposition was made using an air displacement pipette (ADP).
**Table S10:** Precision, accuracy and linearity statistics at each concentration for caffeine analysis in organic solvent (50:50 MeOH:MeCN) with no inclusion of an internal standard (IS) and with melatonin, theobromine and caffeine‐(trimethyl‐^13^C3) (^13^CCaff) as an IS. A 4 μL deposition was made using a positive displacement pipette (PDP).
**Table S11:** Precision, accuracy and linearity statistics at each concentration for caffeine analysis in organic solvent (50:50 MeOH:MeCN) with no inclusion of an internal standard (IS) and with melatonin, theobromine and caffeine‐(trimethyl‐^13^C3) (^13^CCaff) as an IS. A 6 μL deposition was made using an air displacement pipette (ADP).
**Table S12:** Precision, accuracy and linearity statistics at each concentration for caffeine analysis in organic solvent (50:50 MeOH:MeCN) with no inclusion of an internal standard (IS) and with melatonin, theobromine and caffeine‐(trimethyl‐^13^C3) (^13^CCaff) as an IS. A 6 μL deposition was made using a positive displacement pipette (PDP).
**Table S13:** Precision, accuracy and linearity statistics at each concentration for caffeine analysis in organic solvent (50:50 MeOH:MeCN) with no inclusion of an internal standard (IS) and with melatonin, theobromine and caffeine‐(trimethyl‐^13^C3) (^13^CCaff) as an IS. An 8 μL deposition was made using an air displacement pipette (ADP).
**Table S14:** Precision, accuracy and linearity statistics at each concentration for caffeine analysis in organic solvent (50:50 MeOH:MeCN) with no inclusion of an internal standard (IS) and with melatonin, theobromine and caffeine‐(trimethyl‐^13^C3) (^13^CCaff) as an IS. An 8 μL deposition was made using a positive displacement pipette (PDP).
**Table S15:** Precision, accuracy and linearity statistics at each concentration for caffeine analysis in organic solvent (50:50 MeOH:MeCN) with no inclusion of an internal standard (IS) and with melatonin, theobromine and caffeine‐(trimethyl‐^13^C3) (^13^CCaff) as an IS. A 10 μL deposition was made using an air displacement pipette (ADP).
**Table S16:** Precision, accuracy and linearity statistics at each concentration for caffeine analysis in organic solvent (50:50 MeOH:MeCN) with no inclusion of an internal standard (IS) and with melatonin, theobromine and caffeine‐(trimethyl‐^13^C3) (^13^CCaff) as an IS. A 10 μL deposition was made using a positive displacement pipette (PDP).
**Table S17:** Precision, accuracy and linearity statistics at each concentration for caffeine analysis using atmospheric solids analysis probe‐mass spectrometry in organic solvent (50:50 MeOH:MeCN) with no inclusion of an internal standard (IS) and with melatonin, theobromine and caffeine‐(trimethyl‐^13^C3) (^13^CCaff) as an IS. Samples were deposited using a dipping approach.
**Figure S1:** Example simple linear regression calibration curves for caffeine analysis using atmospheric solids analysis probe‐mass spectrometry in organic solvent (50:50 MeOH:MeCN) with caffeine‐(trimethyl‐^13^C3) as an internal standard. Analyses employing a 4 μL deposition volume are shown using (A) an air displacement pipette and (B) a positive displacement pipette, with further visualisation of quantitative linearity using (C) a dipping approach. The symbols refer to the mean measured values with error bars showing the range of values obtained from ten repeated measurements.

## Data Availability

The data that support the findings of this study are available from the corresponding author upon reasonable request.
