# Supplementary material for: Assessing the Quantitative Performance of Atmospheric Solids Analysis Probe‐Mass Spectrometry
Source: Rapid Commun Mass Spectrom. 2025 Aug 1;39(22):e10112. doi: 10.1002/rcm.10112 (PMC12314589; doi:10.1002/rcm.10112)
Supplement: Supplementary file 1 — Table S1: Precision, accuracy and linearity statistics at each concentration for caffeine analysis using atmospheric solids analysis probe‐mass spectrometry in aqueous and organic solvent matrices applying a 2 μL deposition with an air displacement (ADP) and positive displacement (PDP) pipette. Table S2: Precision, accuracy and linearity statistics at each concentration for caffeine analysis using atmospheric solids analysis probe‐mass spectrometry in aqueous and organic solvent matrices applying a 4 μL deposition with an air displacement (ADP) and positive displacement (PDP) pipette. Table S3: Precision, accuracy and linearity statistics at each concentration for caffeine analysis using atmospheric solids analysis probe‐mass spectrometry, in aqueous and organic solvent matrices applying a 6 μL deposition with an air displacement (ADP) and positive displacement (PDP) pipette. Table S6: Precision, accuracy and linearity statistics at each calibration level using the dipping approach for caffeine analysis by atmospheric solids analysis probe‐mass spectrometry. Shaded boxes note concentrations at which no signal was detected. Table S7: Precision, accuracy and linearity statistics at each concentration for caffeine analysis in organic solvent (50:50 MeOH:MeCN) with no inclusion of an internal standard (IS) and with melatonin, theobromine and caffeine‐(trimethyl‐13C3) (13CCaff) as an IS. A 2 μL deposition was made using an air displacement pipette (ADP). Table S8: Precision, accuracy and linearity statistics at each concentration for caffeine analysis in organic solvent (50:50 MeOH:MeCN) with no inclusion of an internal standard (IS) and with melatonin, theobromine and caffeine‐(trimethyl‐13C3) (13CCaff) as an IS. A 2 μL deposition was made using a positive displacement pipette (PDP). Table S9: Precision, accuracy and linearity statistics at each concentration for caffeine analysis in organic solvent (50:50 MeOH:MeCN) with no inclusion of an internal standard (IS) and wi [file RCM-39-e10112-s001.pdf]

SUPPLEMENTARY INFORMATION

Assessing the Quantitative Performance of Atmospheric Solids Analysis Probe-Mass Spectrometry

Alisha Henderson, Stephanie Rankin-Turner, James C Reynolds, Matthew A Turner, Ashley Sage, David Douce, Mario Thevis, Liam M Heaney

Table S1: Precision, accuracy, and linearity statistics at each concentration for caffeine analysis using atmospheric solids analysis probe-mass spectrometry, in aqueous and organic solvent matrices applying a 2 µL deposition with an air displacement (ADP) and positive displacement (PDP) pipette**.**

**[Caffeine]**	**ADP (aq)**		**PDP (aq)**		**ADP (org)**		**PDP (org)**	
**(ng/mL)**	*Precision*	*Accuracy*	*Precision*	*Accuracy*	*Precision*	*Accuracy*	*Precision*	*Accuracy*
50	47%	266%	28%	138%	39%	59%	66%	45%
200	34%	180%	41%	146%	72%	142%	65%	35%
500	54%	164%	34%	53%	65%	62%	74%	47%
1000	73%	179%	28%	123%	62%	70%	56%	44%
2500	51%	127%	27%	77%	36%	47%	85%	35%
5000	22%	89%	44%	105%	35%	115%	106%	26%
	$r^2 = 0.9566$		$r^2 = 0.9840$		$r^2 = 0.9301$		$r^2 = 0.9867$	

Table S2: Precision, accuracy, and linearity statistics at each concentration for caffeine analysis using atmospheric solids analysis probe-mass spectrometry, in aqueous and organic solvent matrices applying a 4 µL deposition with an air displacement (ADP) and positive displacement (PDP) pipette**.**

**[Caffeine]**	**ADP (aq)**		**PDP (aq)**		**ADP (org)**		**PDP (org)**	
**(ng/mL)**	*Precision*	*Accuracy*	*Precision*	*Accuracy*	*Precision*	*Accuracy*	*Precision*	*Accuracy*
50	25%	128%	41%	80%	42%	66%	28%	62%
200	43%	56%	29%	113%	52%	155%	41%	62%
500	67%	31%	47%	38%	29%	79%	34%	60%
1000	38%	82%	18%	100%	44%	47%	28%	53%
2500	95%	36%	21%	112%	35%	85%	27%	77%
5000	29%	118%	28%	98%	50%	106%	44%	108%
	$r^2 = 0.9030$		$r^2 = 0.9939$		$r^2 = 0.9836$		$r^2 = 0.9770$	

Table S3: Precision, accuracy, and linearity statistics at each concentration for caffeine analysis using atmospheric solids analysis probe-mass spectrometry, in aqueous and organic solvent matrices applying a 6 µL deposition with an air displacement (ADP) and positive displacement (PDP) pipette**.**

**[Caffeine]**	**ADP (aq)**		**PDP (aq)**		**ADP (org)**		**PDP (org)**	
**(ng/mL)**	*Precision*	*Accuracy*	*Precision*	*Accuracy*	*Precision*	*Accuracy*	*Precision*	*Accuracy*
50	44%	167%	34%	37%	58%	364%	36%	67%
200	34%	80%	44%	108%	51%	190%	34%	93%
500	31%	28%	28%	28%	31%	109%	17%	76%
1000	67%	87%	23%	90%	53%	103%	71%	90%
2500	48%	151%	27%	132%	41%	71%	23%	56%
5000	30%	89%	30%	93%	31%	107%	30%	112%
	$r^2 = 0.9398$		$r^2 = 0.9730$		$r^2 = 0.9741$		$r^2 = 0.9547$	

Table S4: Precision, accuracy, and linearity statistics at each concentration for caffeine analysis using atmospheric solids analysis probe-mass spectrometry, in aqueous and organic solvent matrices applying a 8 µL deposition with an air displacement (ADP) and positive displacement (PDP) pipette**.**

**[Caffeine]**	**ADP (aq)**		**PDP (aq)**		**ADP (org)**		**PDP (org)**	
**(ng/mL)**	*Precision*	*Accuracy*	*Precision*	*Accuracy*	*Precision*	*Accuracy*	*Precision*	*Accuracy*
50	23%	115%	36%	38%	64%	56%	23%	60%
200	36%	59%	17%	90%	64%	140%	47%	116%
500	25%	22%	48%	28%	58%	85%	36%	84%
1000	65%	56%	21%	98%	80%	87%	61%	86%
2500	11%	186%	16%	159%	23%	60%	43%	79%
5000	21%	81%	23%	86%	31%	111%	43%	106%
	$r^2 = 0.8485$		$r^2 = 0.9214$		$r^2 = 0.9617$		$r^2 = 0.9885$	

Table S5: Precision, accuracy, and linearity statistics at each concentration for caffeine analysis using atmospheric solids analysis probe-mass spectrometry, in aqueous and organic solvent matrices applying a 10 µL deposition with an air displacement (ADP) and positive displacement (PDP) pipette**.**

**[Caffeine]**	**ADP (aq)**		**PDP (aq)**		**ADP (org)**		**PDP (org)**	
**(ng/mL)**	*Precision*	*Accuracy*	*Precision*	*Accuracy*	*Precision*	*Accuracy*	*Precision*	*Accuracy*
50	33%	111%	30%	43%	140%	78%	60%	63%
200	58%	95%	56%	147%	75%	150%	44%	75%
500	25%	20%	28%	20%	39%	60%	42%	102%
1000	35%	82%	28%	114%	87%	91%	28%	112%
2500	42%	191%	11%	144%	27%	40%	26%	53%
5000	29%	79%	22%	89%	31%	116%	64%	111%
	$r^2 = 0.8332$		$r^2 = 0.9514$		$r^2 = 0.9188$		$r^2 = 0.9507$	

Table S6: Precision, accuracy, and linearity statistics at each calibration level using the dipping approach for caffeine analysis by atmospheric solids analysis probe-mass spectrometry. Shaded boxes note concentrations at which no signal was detected.

[Caffeine] (ng/mL)	**Aqueous**		**Organic**	
	Precision	*Accuracy*	*Precision*	*Accuracy*
50				
200			46%	37%
500	34%	52%	31%	45%
1000	44%	115%	27%	22%
2500	20%	251%	31%	8%
5000	58%	62%	36%	40%
	$r^2 = 0.6431$		$r^2 = 0.9505$	

Table S7: Precision, accuracy, and linearity statistics at each concentration for caffeine analysis in organic solvent (50:50 MeOH:MeCN) with no inclusion of an internal standard (IS), and with melatonin, theobromine and caffeine-(trimethyl-$^{13}C_3$) ($^{13}C_{Caff}$) as an internal standard. A 2 µL deposition was made using an air displacement pipette (ADP).

**[Caffeine] (ng/mL)**	**ADP (No IS)**		**ADP (melatonin)**		**ADP (theobromine)**		**ADP ($^{13}C_{Caff}$)**	
	Precision	*Accuracy*	*Precision*	*Accuracy*	*Precision*	*Accuracy*	*Precision*	*Accuracy*
50	39%	59%	51%	123%	77%	82%	62%	181%
200	72%	142%	54%	109%	63%	158%	35%	196%
500	65%	62%	22%	72%	43%	59%	5%	123%
1000	62%	70%	40%	94%	54%	82%	8%	99%
2500	36%	47%	23%	27%	27%	105%	5%	89%
5000	35%	115%	38%	116%	36%	100%	8%	103%
	$r^2 = 0.9301$		$r^2 = 0.8853$		$r^2 = 0.9968$		$r^2 = 0.9956$	

Table S8: Precision, accuracy, and linearity statistics at each concentration for caffeine analysis in organic solvent (50:50 MeOH:MeCN) with no inclusion of an internal standard (IS), and with melatonin, theobromine and caffeine-(trimethyl-$^{13}C_3$) ($^{13}C_{Caff}$) as an internal standard. A 2 µL deposition was made using a positive displacement pipette (PDP)**.**

**[Caffeine]**	**PDP (No IS)**		**PDP (melatonin)**		**PDP (theobromine)**		**PDP ($^{13}C_{Caff}$)**	
**(ng/mL)**	*Precision*	*Accuracy*	*Precision*	*Accuracy*	*Precision*	*Accuracy*	*Precision*	*Accuracy*
50	66%	45%	23%	113%	23%	100%	18%	179%
200	65%	35%	29%	193%	26%	187%	11%	165%
500	74%	47%	30%	77%	33%	67%	12%	117%
1000	56%	44%	28%	84%	62%	100%	6%	98%
2500	85%	35%	13%	27%	20.84	94%	5%	93%
5000	106%	26%	18%	120%	30.37	103%	9%	101%
	$r^2 = 0.9867$		$r^2 = 0.8821$		$r^2 = 0.9969$		$r^2 = 0.9982$	

Table S9: Precision, accuracy, and linearity statistics at each concentration for caffeine analysis in organic solvent (50:50 MeOH:MeCN) with no inclusion of an internal standard (IS), and with melatonin, theobromine and caffeine-(trimethyl-$^{13}C_3$) ($^{13}C_{Caff}$) as an internal standard. A 4 µL deposition was made using an air displacement pipette (ADP)**.**

**[Caffeine]**	**ADP (No IS)**		**ADP (melatonin)**		**ADP (theobromine)**		**ADP ($^{13}C_{Caff}$)**	
**(ng/mL)**	*Precision*	*Accuracy*	*Precision*	*Accuracy*	*Precision*	*Accuracy*	*Precision*	*Accuracy*
50	42%	66%	73%	169%	43%	93%	33%	205%
200	52%	155%	52%	122%	46%	264%	15%	178%
500	29%	79%	26%	84%	27%	67%	13%	128%
1000	44%	47%	38%	97%	34%	102%	6%	102%
2500	35%	85%	24%	34%	30%	112%	4%	99%
5000	50%	106%	26%	115%	25%	97%	11%	100%
	$r^2 = 0.9836$		$r^2 = 0.8853$		$r^2 = 0.9045$		$r^2 = 0.9985$	

Table S10: Precision, accuracy, and linearity statistics at each concentration for caffeine analysis in organic solvent (50:50 MeOH:MeCN) with no inclusion of an internal standard (IS), and with melatonin, theobromine and caffeine-(trimethyl-$^{13}C_3$) ($^{13}C_{Caff}$) as an internal standard. A 4 µL deposition was made using a positive displacement pipette (PDP).

**[Caffeine]**	**PDP (No IS)**		**PDP (melatonin)**		**PDP (theobromine)**		**PDP ($^{13}C_{Caff}$)**	
**(ng/mL)**	*Precision*	*Accuracy*	*Precision*	*Accuracy*	*Precision*	*Accuracy*	*Precision*	*Accuracy*
50	28%	62%	28%	174%	28%	94%	25%	171%
200	41%	62%	39%	127%	26%	326%	10%	173%
500	34%	60%	20%	88%	23%	96%	7%	121%
1000	28%	53%	23%	100%	23%	122%	7%	102%
2500	27%	77%	13%	31%	18%	113%	3%	92%
5000	44%	108%	27%	117%	47%	94%	8%	100%
	$r^2 = 0.977$		$r^2 = 0.8974$		$r^2 = 0.9863$		$r^2 = 0.9979$	

Table S11: Precision, accuracy, and linearity statistics at each concentration for caffeine analysis in organic solvent (50:50 MeOH:MeCN) with no inclusion of an internal standard (IS), and with melatonin, theobromine and caffeine-(trimethyl-$^{13}C_3$) ($^{13}C_{Caff}$) as an internal standard. A 6 µL deposition was made using an air displacement pipette (ADP).

**[Caffeine]**	**ADP (No IS)**		**ADP (melatonin)**		**ADP (theobromine)**		**ADP ($^{13}C_{Caff}$)**	
**(ng/mL)**	*Precision*	*Accuracy*	*Precision*	*Accuracy*	*Precision*	*Accuracy*	*Precision*	*Accuracy*
50	58%	364%	31%	174%	29%	140%	11%	189%
200	51%	190%	30%	162%	43%	362%	16%	165%
500	31%	109%	68%	158%	40%	109%	8%	144%
1000	53%	103%	24%	123%	33%	101%	9%	111%
2500	41%	71%	18%	43%	24%	104%	11%	107%
5000	31%	107%	24%	112%	22%	98%	9%	97%
	$r^2 = 0.9741$		$r^2 = 0.9271$		$r^2 = 0.9908$		$r^2 = 0.9961$	

Table S12: Precision, accuracy, and linearity statistics at each concentration for caffeine analysis in organic solvent (50:50 MeOH:MeCN) with no inclusion of an internal standard (IS), and with melatonin, theobromine and caffeine-(trimethyl-$^{13}C_3$) ($^{13}C_{Caff}$) as an internal standard. A 6 µL deposition was made using a positive displacement pipette (PDP).

**[Caffeine]**	**PDP (No IS)**		**PDP (melatonin)**		**PDP (theobromine)**		**PDP ($^{13}C_{Caff}$)**	
**(ng/mL)**	*Precision*	*Accuracy*	*Precision*	*Accuracy*	*Precision*	*Accuracy*	*Precision*	*Accuracy*
50	36%	67%	25%	176%	17%	131%	8%	196%
200	34%	93%	24%	153%	27%	326%	13%	204%
500	17%	76%	23%	120%	31%	76%	15%	127%
1000	71%	90%	80%	160%	26%	167%	4%	118%
2500	23%	56%	21%	36%	24%	93%	4%	102%
5000	30%	112%	20%	112%	20%	97%	8%	98%
	$r^2 = 0.9547$		$r^2 = 0.9071$		$r^2 = 0.9778$		$r^2 = 0.9967$	

Table S13: Precision, accuracy, and linearity statistics at each concentration for caffeine analysis in organic solvent (50:50 MeOH:MeCN) with no inclusion of an internal standard (IS), and with melatonin, theobromine and caffeine-(trimethyl-$^{13}C_3$) ($^{13}C_{Caff}$) as an internal standard. An 8 µL deposition was made using an air displacement pipette (ADP).

**[Caffeine]**	**ADP (No IS)**		**ADP (melatonin)**		**ADP (theobromine)**		**ADP ($^{13}C_{Caff}$)**	
**(ng/mL)**	*Precision*	*Accuracy*	*Precision*	*Accuracy*	*Precision*	*Accuracy*	*Precision*	*Accuracy*
50	64%	56%	25%	215%	26%	147%	11%	241%
200	64%	140%	53%	16%	40%	348%	10%	203%
500	58%	85%	37%	161%	49%	113%	44%	126%
1000	80%	87%	51%	172%	49%	122%	17%	107%
2500	23%	60%	11%	40%	15%	99%	5%	108%
5000	31%	111%	25%	112%	22%	98%	8%	100%
	$r^2 = 0.9617$		$r^2 = 0.9093$		$r^2 = 0.9904$		$r^2 = 0.9970$	

Table S14: Precision, accuracy, and linearity statistics at each concentration for caffeine analysis in organic solvent (50:50 MeOH:MeCN) with no inclusion of an internal standard (IS), and with melatonin, theobromine and caffeine-(trimethyl-$^{13}C_3$) ($^{13}C_{Caff}$) as an internal standard. An 8 µL deposition was made using a positive displacement pipette (PDP).

**[Caffeine]**	**PDP (No IS)**		**PDP (melatonin)**		**PDP (theobromine)**		**PDP ($^{13}C_{Caff}$)**	
**(ng/mL)**	*Precision*	*Accuracy*	*Precision*	*Accuracy*	*Precision*	*Accuracy*	*Precision*	*Accuracy*
50	23%	60%	8%	187%	17%	149%	14%	210%
200	47%	116%	26%	162%	30%	475%	7%	201%
500	36%	84%	17%	180%	44%	92%	12%	140%
1000	61%	86%	15%	206%	45%	84%	4%	114%
2500	43%	79%	14%	39%	23%	115%	3%	100%
5000	43%	106%	42%	115%	23%	95%	10%	100%
	$r^2 = 0.9885$		$r^2 = 0.8931$		$r^2 = 0.9756$		$r^2 = 0.9969$	

Table S15: Precision, accuracy, and linearity statistics at each concentration for caffeine analysis in organic solvent (50:50 MeOH:MeCN) with no inclusion of an internal standard (IS), and with melatonin, theobromine and caffeine-(trimethyl-$^{13}C_3$) ($^{13}C_{Caff}$) as an internal standard. A 10 µL deposition was made using an air displacement pipette (ADP).

**[Caffeine]**	**ADP (No IS)**		**ADP (melatonin)**		**ADP (theobromine)**		**ADP ($^{13}C_{Caff}$)**	
**(ng/mL)**	*Precision*	*Accuracy*	*Precision*	*Accuracy*	*Precision*	*Accuracy*	*Precision*	*Accuracy*
50	140%	78%	19%	248%	26%	191%	18%	233%
200	75%	150%	37%	236%	31%	424%	24%	178%
500	39%	60%	20%	163%	62%	1539%	19%	151%
1000	87%	91%	54%	162%	31%	131%	4%	114%
2500	27%	40%	20%	47%	23%	95%	16%	95%
5000	31%	116%	33%	117%	20%	86%	13%	101%
	$r^2 = 0.9188$		$r^2 = 0.9253$		$r^2 = 0.3837$		$r^2 = 0.9961$	

Table S16: Precision, accuracy, and linearity statistics at each concentration for caffeine analysis in organic solvent (50:50 MeOH:MeCN) with no inclusion of an internal standard (IS), and with melatonin, theobromine and caffeine-(trimethyl-$^{13}C_3$) ($^{13}C_{Caff}$) as an internal standard. A 10 µL deposition was made using a positive displacement pipette (PDP).

**[Caffeine]**	**PDP (No IS)**		**PDP (melatonin)**		**PDP (theobromine)**		**PDP ($^{13}C_{Caff}$)**	
**(ng/mL)**	*Precision*	*Accuracy*	*Precision*	*Accuracy*	*Precision*	*Accuracy*	*Precision*	*Accuracy*
50	60%	63%	12%	206%	17%	157%	8%	211%
200	44%	75%	34%	165%	38%	364%	7%	187%
500	42%	102%	21%	161%	28%	74%	21%	93%
1000	28%	112%	26%	127%	29%	105%	11%	99%
2500	26%	53%	14%	43%	23%	104%	6%	98%
5000	64%	111%	25%	112%	20%	100%	12%	99%
	$r^2 = 0.9507$		$r^2 = 0.9263$		$r^2 = 0.991$		$r^2 = 0.9989$	

Table S17: Precision, accuracy, and linearity statistics at each concentration for caffeine analysis using atmospheric solids analysis probe-mass spectrometry in organic solvent (50:50 MeOH:MeCN) with no inclusion of an internal standard (IS), and with melatonin, theobromine and caffeine-(trimethyl-$^{13}C_3$) ($^{13}C_{Caff}$) as an internal standard. Samples were deposited using a dipping approach.

**[Caffeine]**	**No IS**		**Melatonin IS**		**Theobromine IS**		**$^{13}C_{Caff}$ IS**	
**(ng/mL)**	*Precision*	*Accuracy*	*Precision*	*Accuracy*	*Precision*	*Accuracy*	*Precision*	*Accuracy*
500	31%	45%	16%	79%	30%	41%	17%	128%
1000	27%	22%	32%	46%	50%	26%	32%	86%
2500	31%	8%	46%	28%	40%	79%	23%	90%
5000	36%	40%	32%	122%	32%	108%	16%	101%
	$r^2 = 0.9505$		$r^2 = 0.8755$		$r^2 = 0.9684$		$r^2 = 0.9969$	

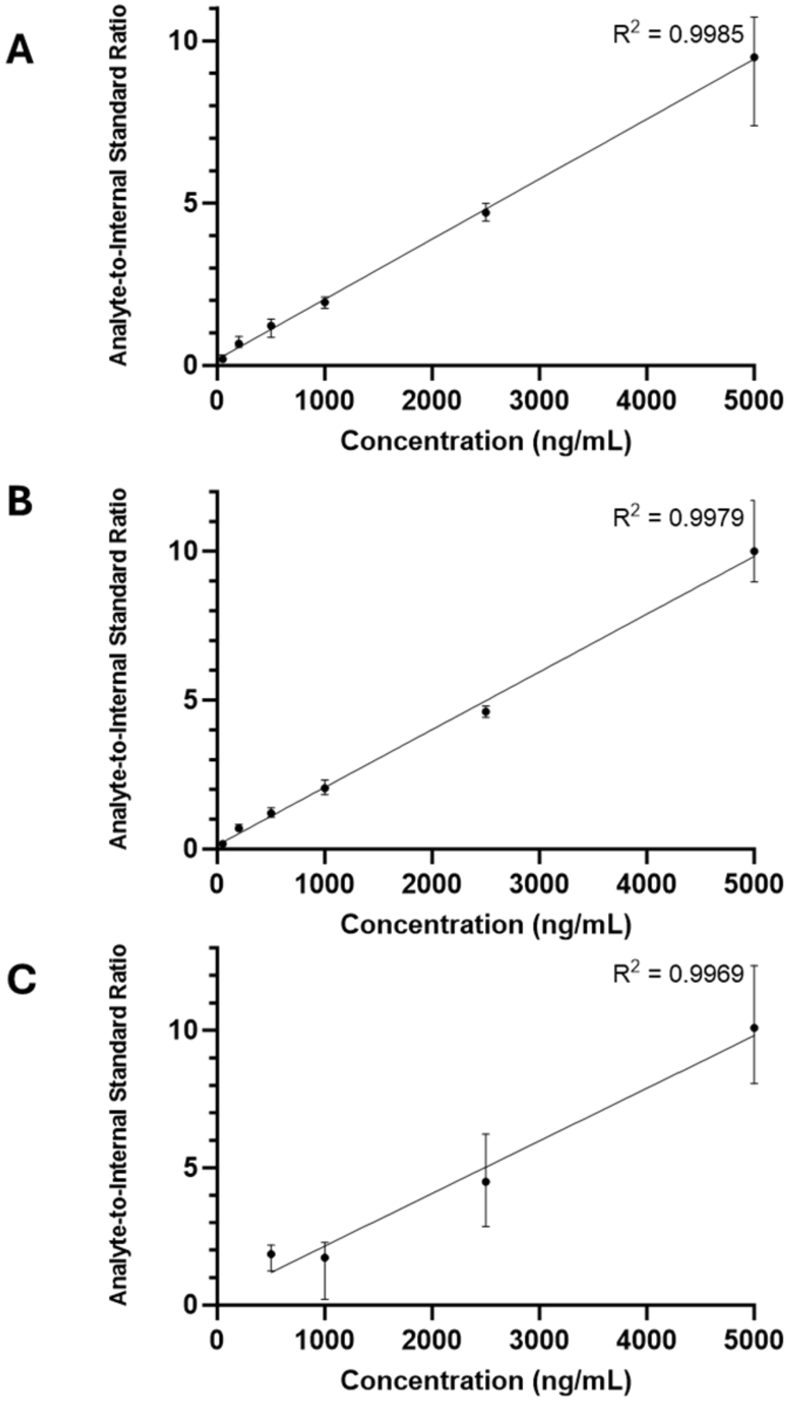

Figure S1: Example simple linear regression calibration curves for caffeine analysis using atmospheric solids analysis probe-mass spectrometry, in organic solvent (50:50 MeOH:MeCN) with caffeine-(trimethyl-$^{13}C_3$) as an internal standard. Analyses employing a 4 µL deposition volume are shown using (**A**) an air displacement pipette and (**B**) a positive displacement pipette, with further visualisation of quantitative linearity using (**C**) a dipping approach. The symbols refer to the mean measured values with error bars showing the range of values obtained from ten repeated measurements.
